# Validation of the eighth edition of the AJCC staging system for patients with pancreatic adenocarcinoma initially receiving chemoradiotherapy and proposal of modifications

**DOI:** 10.20892/j.issn.2095-3941.2019.0101

**Published:** 2020-05-15

**Authors:** Xiaofei Zhu, Di Chen, Yangsen Cao, Xianzhi Zhao, Xiaoping Ju, Yuxin Shen, Fei Cao, Shuiwang Qing, Fang Fang, Zhen Jia, Huojun Zhang

**Affiliations:** ^1^Department of Radiation Oncology, Changhai Hospital Affiliated to Navy Medical University, Shanghai 200433, China

**Keywords:** Chemotherapy, modifications, pancreatic cancer, stereotactic body radiation therapy, the eighth edition of the AJCC staging system

## Abstract

**Objective:** To validate the eighth edition of the AJCC staging system in patients with pancreatic adenocarcinoma receiving only stereotactic body radiation therapy and chemotherapy, and to propose modifications to improve prognostic accuracy.

**Methods:** Patients with pathologically confirmed pancreatic adenocarcinoma without metastasis who were undergoing only chemoradiotherapy were included and staged according to the seventh and eighth editions of the AJCC staging system. Meanwhile, another group of stage T4 patients from the above enrollment with only portal vein involvement with or without tumor thrombi (PV ± PVTT) were retrieved for survival comparisons. Modifications were proposed according to the survival comparisons. A cohort from the SEER database was used for external validation of the modified staging system.

**Results:** A total of 683 patients were included. Patients with N2 or N1 but different T stages had significantly different survival outcomes according to the eighth edition. The survival of patients with PV ± PVTT was comparable to that of patients with T4 tumors. The concordance index of the seventh and eighth editions, and the modified staging system was 0.744 (95%CI: 0.718–0.769), 0.750 (95%CI: 0.725–0.775), and 0.788 (95%CI: 0.762–0.813), respectively. For external validation, the concordance index was 0.744 (95%CI: 0.718–0.770), 0.750 (95%CI: 0.724–0.776), and 0.788 (95%CI: 0.762–0.814), respectively.

**Conclusions:** The modified staging system is suggested to have the most accurate prognostic value. Hence, PV ± PVTT should be added to the definition of T4 tumors regardless of tumor size. Patients with N2 or N1 in different T stages could be regrouped into different substages. Additionally, stage III should be subclassified into IIIA (T3N2 and T4N0) and IIIB (T4N1-2).

## Introduction

In pancreatic cancer, multimodal treatment based on prognostic assessment is crucial. Prognostic assessment, such as survival estimation, relies on the accuracy of staging systems, which contribute to decision-making in individualized treatment as well as the evaluation of treatment outcomes. Accurate staging systems are also informative to patients regarding their prognoses.

Previously, the seventh edition of the AJCC staging system was criticized for its poor performance in clinical practice and non-specific T stages^1^. The 8th edition introduced 2 major modifications. First, the T3 category is determined by tumor size rather than extrapancreatic invasion. Because of the lack of a capsule, distinguishing the pancreas from extrapancreatic tissues is difficult, thus potentially resulting in irreproducible T staging^1^. Second, N1 is sub-stratified into N1 (1–3 positive regional lymph nodes) and N2 (≥ 4 positive lymph nodes), given that the number and ratio of positive lymph nodes are predictive of patient survival^2–4^.

Although validation studies for the eighth edition implied greater prognostic accuracy than that of the seventh edition^5–7^, the new edition has been validated only in patients with resectable or borderline resectable pancreatic cancer receiving surgery. However, no studies have confirmed the accurate survival prediction of the eighth edition for patients initially treated with chemoradiotherapy without surgery, and the majority of patients in the validation studies had advanced-stage pancreatic cancer at the initial diagnosis and chemoradiotherapy as the first option^8^. Hence, the aim of this study was to evaluate the prognostic value of the eighth edition of the AJCC staging system for patients with stereotactic body radiation therapy (SBRT) and chemotherapy. Furthermore, potential modifications are proposed for the improvement of the eighth edition.

## Materials and methods

### Patients

The study was approved by the institutional review board of Changhai Hospital Affiliated to Navy Medical University (Approval No. 2016-CHYY-072). Imaging examinations including CT, MRI, and PET-CT were required for stage determination before treatment. Additionally, biopsies with fine needle aspiration guided by endoscopic ultrasound were mandatory. Moreover, the medical records of patients with resectable or borderline resectable pancreatic cancer were first reviewed by surgeons. SBRT and chemotherapy were initiated only if cases were medially inoperable because of comorbidities or patients refused surgical resection. Patients with distant metastasis or those receiving surgical resection were excluded. Therefore, the enrolled patients all had pathologically and radiographically confirmed resectable, borderline resectable, or locally advanced pancreatic cancer.

Another cohort was retrieved from the Surveillance, Epidemiology, and End Results (SEER) database (from 1973 to 2015) to externally evaluate the modified staging system. Data were collected according to the site codes (C25.0–C25.4 and C25.7–C25.9) of the International Classification of Disease 3rd edition (ICD-O3). For confirmation of pancreatic adenocarcinoma or infiltrating ductal carcinoma, the ICD-O-3 histology/behavior codes of 8500/3 and 8140/3 were used. Additionally, only patients receiving chemoradiotherapy without surgery and with confirmed tumor sizes and numbers of positive lymph nodes were included.

### Delivery of SBRT

SBRT was performed with protocols described in our previous studies^9–11^ with a CyberKnife^®^ (Accuray Incorporated, Sunnyvale, CA, USA). Fiducial makers were implanted for image guidance, and a minimum of 3 were inserted within or adjacent to the tumor. Gross tumor volume (GTV) was defined by gross disease identified radiographically. The clinical target volume was equal to the GTV in most cases. The planning target volume was determined with a 2–5 mm margin expansion from the GTV at the physician’s discretion. The dose constraints of organs at risk were based on the American Association of Physicists in Medicine guidelines in TG-101^12^. Delineations of target volumes were reviewed and verified by a radiation oncologist and a radiologist. Triphasic enhanced CT images were acquired for contours.

### Chemotherapy regimens

Chemotherapy followed by SBRT was the initial therapy, which was performed 2 to 3 weeks after completion of SBRT. The regimen included gemcitabine and S-1. Previous studies have indicated that the survival benefits of S-1 are comparable to those of gemcitabine without increasing the incidence of toxicity^13–15^. Gemcitabine (1000 mg/m^2^) was delivered on days 1, 8, and 15 during a 4-week cycle, which continued for 4 to 6 cycles. S-1 was given orally at a dose of 80 mg/m^2^ for 28 days and was followed by a 14-day rest with 4 to 6 cycles.

### Cancer staging

Patients were staged according to the seventh and eighth edition staging systems. Patients with undefined cancer stage due to missing data were excluded. The tumor diameter was the maximum diameter measured in imaging examinations. Regional lymph nodes along the lymphatic drainage sites in the surgical field were included in the N categories^16^. The evaluation of cancer stages was performed by 2 radiologists. Consensus agreement was achieved with regard to any disagreement between the observers.

### Statistical analysis

Continuous data are displayed as medians and ranges. Categorical data are presented as frequencies. Proportions were compared with chi-square tests. Overall survival (OS) was determined between the date of SBRT and the date of death or last follow-up, and was calculated and compared with the Kaplan-Meier method and log-rank tests. The Cox proportional hazards model was used to assess the effects of different cancer stages on OS.

The prognostic accuracy of the seventh and eighth editions in terms of OS was evaluated with the concordance index (C index), receiver operating characteristic (ROC) curve, and time-dependent area under the curve (AUC)^17–19^. Additionally, prognosis estimates based on the proposed modified staging system were further compared with those based on the seventh and eighth editions *via* the C index, AUC, and net reclassification index (NRI)^17–19^. Because the ROC curve and NRI were used to assess the quantification of survival of models for a fixed moment in time, time points 1 and 2 years after treatment (1-year and 2-year survival) were chosen for the analysis. External validation was performed according to the C index and AUC. Statistical analyses were performed in SPSS version 22.0 (IBM Corporation, Armonk, NY, USA) and R 3.5.1. Two-sided *P* values < 0.05 were considered statistically significant.

## Results

### Patient characteristics

The baseline patient characteristics are shown in **Table 1**. A total of 683 patients were included. The median follow-up was 15.6 months (range: 2.6–45.5 months). The median prescription dose and biological effective dose (BED10, α/β = 10) was 37 Gy (range: 32.5–49.6 Gy) in 5–8 fractions and 61.92 Gy (53.625–88.32 Gy) in 5–8 fractions. After re-classification with the eighth edition of staging system, the cancer stages of 152 patients (22.2%) were revised; 22 (3.2%) patients were assigned to a lower stage, whereas 130 (19.0%) were assigned to a higher stage (**Supplementary Table S1**).

### Survival outcomes according to the seventh and eighth edition staging systems

**Figure 1:**
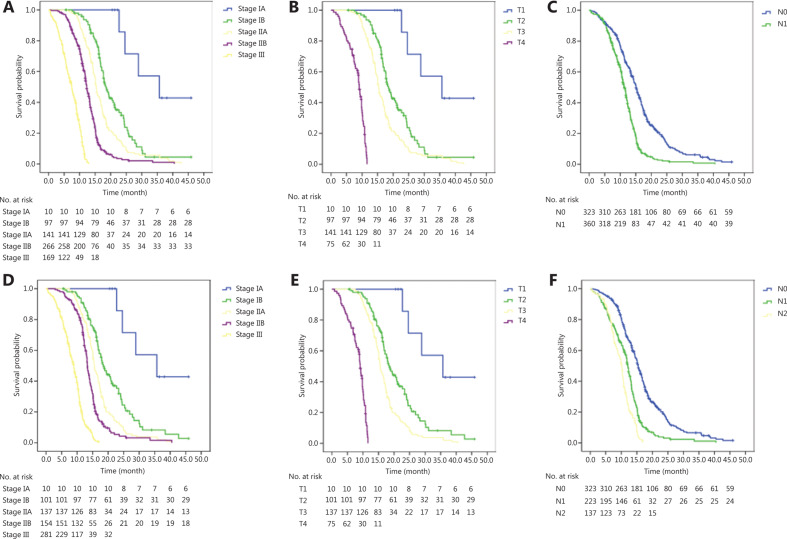
(A) Patient OS according to the seventh edition, (B) the T stage of the seventh edition for patients with negative lymph nodes, (C) the N stage of the seventh edition, (D) patient OS according to the eighth edition, (E) the T stage according to the eighth edition for patients with negative lymph nodes, and (F) the N stage according to the eighth edition.

The median OS of the cohort was 12.7 months (95%CI: 12.1–13.3 months). The 1-year and 2-year OS rates were 55.9% and 19.0%, respectively. The OS curves based on the seventh and eighth editions are shown in **Figure 1A** and **1D**, respectively. Significantly different OS outcomes were found in patients staged by the seventh (*P* < 0.001) and eighth (*P* < 0.001) editions. In sub-group analysis, both T and N stage in the seventh and eighth editions were discriminative for survival (**Figure 1B, 1C, 1E**, and **1F** and **Table 2**).

Multivariate analysis according to the eighth edition showed that advanced T stage and N stage were both associated with poor survival. Compared with that of T1 tumors, the hazard ratios of T2, T3, and T4 tumors were 2.01 (95%CI: 1.21–3.32), 3.81 (95%CI: 2.33–6.25), and 25.34 (95%CI: 14.87–43.20), respectively (*P* < 0.001). Similarly, compared with that of N0, the hazard ratios of N1 and N2 were 2.19 (95%CI: 1.81–2.66) and 3.70 (95%CI: 2.92–4.68), respectively (*P* < 0.001).

### Modification of T stage

Given that tumor invasions of the portal vein with or without a tumor thrombus alone (PV ± PVTT) may be identified at initial diagnosis in some patients, but no details of this aspect are considered in the T staging of the eighth edition, our analysis included another 92 patients with the manifestations for receiving SBRT and chemotherapy (gemcitabine+S-1). Because of the portal vein involvement in the tumors, we compared the survival outcome of patients receiving PV ± PVTT with that of patients with T4 tumors. We found no significant differences in the baseline characteristics of patients between these groups (**Supplementary Table S2**). The median OS of patients with PV ± PVTT and those with T4 tumors was 7.4 months (95%CI: 6.1–8.7 months) and 7.5 months (95%CI: 6.6–8.4 months), respectively (*P* = 0.134) (**Supplementary Figure S1**).

### Modification of the eighth edition of AJCC staging system

**Figure 2:**
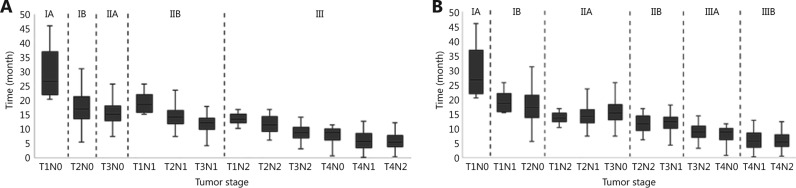
(A) Patient OS in each substage classified according to the eighth edition and (B) the modified staging system.

To investigate whether the eighth edition is discriminative for OS, we compared the survival outcomes for each substage (**Figure 2A**). Within stage IIB, both the longest and shortest OS were found in T1N1 and T3N1, respectively (*P* < 0.001). Similar findings were also identified in the OS of T1N2, T2N2, and T3N2 (*P* < 0.001). Additionally, the median OS of T1N2 was longer than that of T3N1 (*P* = 0.029). Meanwhile, for stage III, the median OS of T3N2 was longer than those of T4N1 and T4N2 (T3N2 *vs.* T4N1, *P* < 0.001; T3N2 *vs.* T4N2, *P* = 0.001). Moreover, superior OS was also found in T4N0 compared with T4N1 and T4N2 (T4N0 *vs.* T4N1, *P* = 0.02; T4N0 *vs.* T4N2, *P* = 0.036) (**Table 3**).

Furthermore, no difference was found in OS among T1N1 and T2N0 (*P* = 0.525), T2N2 and T3N1 (*P* = 0.897), and T3N2 and T4N0 (*P* = 0.122). In addition, similar OS was observed among T1N2, T2N1, and T3N0 (T1N2 *vs.* T2N1, *P* = 0.481; T1N2 *vs.* T3N0, *P* = 0.210; T2N1 *vs.* T3N0, *P* = 0.445) (**Table 3**).

Therefore, the eighth edition may not provide sufficiently accurate and reliable survival estimates. Consequently, on the basis of the definitions for T and N stages in the eighth edition, we propose a modified staging system for the OS of each substage (**Figure 2B**). In contrast to the eighth edition classification, stage III was divided into IIIA (T3N2 and T4N0) and IIIB (T4N1 and T4N2). Moreover, T1N1 and T2N0 were grouped in stage IB, and T1N2, T2N1, and T3N0 were allocated to stage IIA. In addition, the modified stage IIB included T2N2 and T3N1.

On the basis of the modified staging system, the median OS for stages IA, IB, IIA, IIB, IIIA, and IIIB was 35.7 months (95%CI: 18.2–53.2 months), 18.7 months (95%CI: 17.2–20.2 months), 15.4 months (95%CI: 15.1–15.7 months), 12.3 months (95%CI: 11.6–13.0 months), 9.1 months (95%CI: 8.4–9.8 months), and 5.8 months (95%CI: 5.0–6.6 months), respectively (*P* < 0.001) (**Supplementary Figure S2**).

### Prognostic accuracy

The C index for the seventh edition, eighth edition, and modified staging system was 0.744 (95%CI: 0.718–0.769), 0.750 (95%CI: 0.725–0.775), and 0.788 (95%CI: 0.762–0.813), respectively. The ROC curve at 1 year after treatment showed that the AUC for survival according to the seventh edition, eighth edition, and modified staging system was 0.807 (95%CI: 0.775–0.840), 0.814 (95%CI: 0.782–0.847), and 0.850 (95%CI: 0.821–0.880), respectively. Similarly, for 2-year survival, the AUC according to the seventh edition, eighth edition, and modified staging system was 0.680 (95%CI: 0.624–0.735), 0.679 (95%CI: 0.623–0.735), and 0.714 (95%CI: 0.662–0.766), respectively (**Supplementary Figure S3**).

Moreover, all patients had a known vital status at 1 year and 2 years after SBRT and chemotherapy. For 1-year survival, 84 of 300 patients (28.0%) were correctly reclassified into a higher stage, and 23 of 383 patients (6.0%) were correctly reclassified into a lower stage when the modified staging system was applied, as compared with the eighth edition classification. The additive NRI and absolute NRI were 22.51 and 10.4%, respectively. Similarly, for 2-year survival, 87 of 555 patients (15.7%) were correctly reclassified into a higher stage, and 12 of 128 patients (9.4%) were correctly reclassified into a lower stage, as compared with the eighth edition classification. The additive NRI and absolute NRI were 9.74 and 2.0%, respectively.

### External validation of the modified staging system with a cohort from the SEER database

**Figure 3:**
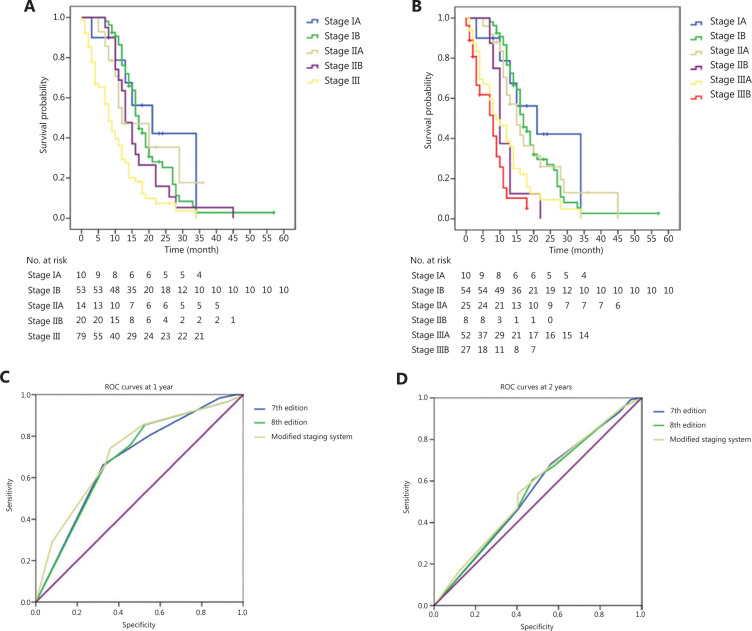
(A) OS of the cohort from the SEER database according to the eighth edition and (B) the modified staging system, (C) ROC curves of the seventh and eighth editions, and the modified staging system at 1 year after treatment and (D) at 2 years after treatment.

The patient characteristics of the cohort are shown in **Supplementary Table S3**. Details of survival are shown in **Figure 3A** and **3B** and **Supplementary Table S4**. The C index for the seventh edition, eighth edition, and modified staging system was 0.744 (95%CI: 0.718–0.770), 0.750 (95%CI: 0.724–0.776), and 0.788 (95%CI: 0.762–0.814), respectively. For 1-year survival, the AUC according to the seventh edition, eighth edition, and modified staging system was 0.686 (95%CI: 0.606–0.767), 0.691 (95%CI: 0.610–0.771), and 0.722 (95%CI: 0.643–0.801), respectively. For 2-year survival, the AUC according to the seventh edition, eighth edition, and modified staging system was 0.555 (95%CI: 0.463–0.648), 0.557 (95%CI: 0.465–0.650) and 0.567 (95%CI: 0.476–0.659), respectively (**Figure 3C** and **3D**).

## Discussion

This pilot study aimed to evaluate the prognostic value of the eighth edition for patients initially treated with SBRT and chemotherapy without surgery. Because of the crucial effects of different treatment modalities and stage distributions on survival, the results of this study may be complementary to those of previous studies focusing on operative patients with early stage pancreatic cancer.

Despite the improvement in T stage, discrepancies in validation results have been found among studies. A recent study has reported that the pT stage of the eighth edition improves prognostication^20^. Nevertheless, patients with positive lymph nodes were included in the analysis, thus potentially leading to overinterpretation of the discrimination of survival by pT stage. In addition, similar results have been described in Allen et al.^6^ However, in that study, only patients with R0 resection were included, and therefore selection bias might have resulted, given that positive margins should be also included in the clinical practice of the staging system. Nevertheless, poor survival discrimination has been found in an international cohort^5^. The difference might be attributable to the variable treatment schemes beyond limitations. Various adjuvant therapies were used in the international cohort, whereas most patients received only adjuvant chemotherapy in these 2 studies. In our study, T stage was found to be correlated with survival; this result might be attributable to the different treatment and stage distributions from those in previous studies. Hence, whether the difference can be ascribed to the different treatment modalities, measurement variability, or the parameter itself may remain controversial.

Furthermore, tumors with involvement of the celiac axis, the superior mesenteric artery, and/or the common hepatic artery have been clarified to be T4. Nevertheless, some patients may have only PV ± PVTT. Given the vascular involvement, determining the T stage only on the basis of tumor size might be inaccurate and thus contribute to overestimation of survival if patients are grouped into the same stages as those only with small tumor sizes. Therefore, in this study, the OS of patients with PV ± PVTT was compared with that of patients with T4 tumors. Similar prognosis was found between these groups, possibly because the patients were at high risk of liver or lung metastasis regarding portal vein invasion, particularly with tumor thrombi. Consequently, PV ± PVTT should be staged as T4 irrespective of tumor size.

Although the improved prognostic value of the eighth edition had been demonstrated, there was still lack of a clear discrimination of survival between substages, as demonstrated by our results. Therefore, according to the survival duration of each substage, we propose a modified staging system. Notably, the greater C index, NRI, and larger AUC of the modified staging system compared with the eighth edition indicated improved prognostic accuracy. Moreover, our results are in line with findings from Song et al.^21^, who have demonstrated superior survival of patients with N2 compared with T4. Our results suggest that stage III should be subdivided into stage IIIA (T1-3N2) and IIIB (T4Nany). However, in this study, patients with N2 regardless of T stage were included for comparison, and we were unable to identify the potential differences in survival between subgroups of N2. Our study revealed that prognosis may differ among N2 patients with different T stages, as well as N1 patients with different T stages. This conclusion is similar to the proposed modification described by Shi et al.^7^ Hence, the selected N2 patients with early T stage, after multidisciplinary approaches, may have a chance of receiving curative surgical resection, whereas patients with T3N2 may be potential candidates for chemoradiotherapy, because they were grouped into the same stage as T4.

Compared with the proposed modification by Shi et al.^7^, T4N0 was regrouped as stage IIIA rather than stage IIIB in our study for 2 reasons. First, because the patients in Shi et al.^7^ all received surgical resection, different treatment modalities should be taken into account. Second, although the patient survival durations in our study were slightly shorter than those in Shi et al.^7^, the performance statuses of patients undergoing curative surgical resection with neoadjuvant or adjuvant therapy were usually much better than those of patients not amenable to surgery with the same cancer stages receiving chemoradiotherapy. In addition, more patients were above 65 years of age in our study than in Shi et al. (55.9% *vs.* 36.8%). Therefore, these 2 factors may have profoundly influenced the survival outcomes.

After external evaluation, the most reliable prediction of survival was also achieved with the modified staging system than with the seventh and eighth editions. However, some confounding factors were inevitable, owing to the limited number of patients and the heterogeneity of the cohort from the SEER database, in which patients were enrolled from 1973 to 2015. Substantial differences in treatment modality and radiographic assessment may have occurred over that time period.

There are several limitations in our study. First, the interpretation of our results might be limited by the retrospective nature of this study. Therefore, the modifications should be used cautiously. Second, owing to a lack of uniform modality and sufficient patients in the cohort from the SEER database, our results should be further validated with a large multi-institutional database prospectively.

## Conclusions

The pilot study validated the eighth edition in patients receiving only chemoradiotherapy, in comparison to the operative patients with early stage pancreatic cancer in previous studies. The survival of patients with PV ± PVTT was comparable to those with T4 tumors, thus indicating that PV ± PVTT might be added to the definition of T4 tumors regardless of tumor size. Additionally, patients with N2 or N1 but different T stages should be grouped into different substages, owing to the differences in OS. The modified staging system might provide a basis for the next AJCC staging systems, and further validation is needed.

## Figures and Tables

**Table 1:** Patient characteristics

Characteristics	*n* (%)
Number of patients	683
Age, years, median (range)	66 (29–90)
Gender	
Male	409 (59.9%)
Female	174 (40.1%)
Tumor diameter (cm), median (range)	3.6 (0.6–9.0)
TNM stage, seventh edition	
IA	10 (1.5%)
IB	97 (14.2%)
IIA	141 (20.6%)
IIB	266 (38.9%)
III	169 (24.7%)
TNM stage, eighth edition	
IA	10 (1.5%)
IB	101 (14.8%)
IIA	137 (20.1%)
IIB	154 (22.5%)
III	281 (41.1%)
Prescription dose (Gy), median (range)	37 (32.5–49.6)/5-8f
BED10 (Gy), median (range)	61.92 (53.625–88.32)/5-8f

**Table 2:** Survival outcomes according to the seventh and eighth edition staging systems

		Seventh edition	*P*	Eighth edition	*P*
		OS (95%CI)		OS (95%CI)	
Cancer stage	IA	35.7 m (95%CI: 18.3–53.2 m)	< 0.001	35.7 m (95%CI: 18.3–53.2 m)	< 0.001
	IB	18.5 m (95%CI: 16.9–20.0 m)		18.5 m (95%CI: 16.8–20.2 m)	
	IIA	15.4 m (95%CI: 14.8–16.0 m)		15.5 m (95%CI: 14.6–16.4 m)	
	IIB	12.3 m (95%CI: 11.7–12.9 m)		13.3 m (95%CI: 12.8–13.8 m)	
	III	7.5 m (95%CI: 6.6–8.4 m)		8.9 m (95%CI: 8.2–9.6 m)	
T stage with N0	T1	35.7 m (95%CI: 18.3–53.2 m)	< 0.001	35.7 m (95%CI: 18.3–53.2 m)	< 0.001
	T2	18.5 m (95%CI: 16.9–20.0 m)		18.5 m (95%CI: 16.8–20.2 m)	
	T3	15.4 m (95%CI: 14.8–16.0 m)		15.5 m (95%CI: 14.6–16.4 m)	
	T4	9.1 m (95%CI: 8.6–9.6 m)		9.1 m (95%CI: 8.6–9.6 m)	
N stage	N0	15.4 m (95%CI: 14.4–16.4 m)	< 0.001	15.4 m (95%CI: 14.4–16.4 m)	< 0.001
	N1	11.0 m (95%CI: 10.4–11.6 m)		12.1 m (95%CI: 11.6–12.6 m)	
	N2	–		10.1 m (95%CI: 9.4–10.8 m)	

**Table 3:** Survival comparisons of substages according to the eighth edition

Stage		OS (95%CI)	*P*
IIB (T1-3N1)	T1N1 *vs.* T2N1	20.9 m (95%CI: 17.5–24.3 m) *vs.* 15.1 m (95%CI: 14.3–15.9 m)	0.011
	T1N1 *vs.* T3N1	20.9 m (95%CI: 17.5–24.3 m) *vs.* 12.3 m (95%CI: 11.6–13.0 m)	< 0.001
	T2N1 *vs.* T3N1	15.1 m (95%CI: 14.3–15.9 m) *vs.* 12.3 m (95%CI: 11.6–13.0 m)	< 0.001
III (T1-3N2)	T1N2 *vs.* T2N2	14.9 m (95%CI: 13.1–16.7 m) *vs.* 11.9 m (95%CI: 11.2–12.6 m)	0.042
	T1N2 *vs.* T3N2	14.9 m (95%CI: 13.1–16.7 m) *vs.* 9.5 m (95%CI: 8.5–10.5 m)	< 0.001
	T2N2 *vs.* T3N2	11.9 m (95%CI: 11.2–12.6 m) *vs.* 9.5 m (95%CI: 8.5–10.5 m)	< 0.001
III (T3N2, T4N1-2)	T3N2 *vs.* T4N0	9.5 m (95%CI: 8.5–10.5 m) *vs.* 9.1 m (95%CI: 8.6–9.6 m)	0.122
	T3N2 *vs.* T4N1	9.5 m (95%CI: 8.5–10.5 m) *vs.* 5.9 m (95%CI: 5.0–6.8 m)	< 0.001
	T3N2 *vs.* T4N2	9.5 m (95%CI: 8.5–10.5 m) *vs.* 5.8 m (95%CI: 5.0–6.6 m)	0.001
	T4N0 *vs.* T4N1	9.1 m (95%CI: 8.6–9.6 m) *vs.* 5.9 m (95%CI: 5.0–6.8 m)	0.02
	T4N0 *vs.* T4N2	9.1 m (95%CI: 8.6–9.6 m) *vs.* 5.8 m (95%CI: 5.0–6.6 m)	0.036
–	T1N1 (IIB) *vs.* T2N0 (IB)	20.9 m (95%CI: 17.5–24.3 m) *vs.* 18.5 m (95%CI: 16.8–20.2 m)	0.525
–	T2N2 (III) *vs.* T3N1 (IIB)	11.9 m (95%CI: 11.2–12.6 m) *vs.* 12.3 m (95%CI: 11.6–13.0 m)	0.897
–	T1N2 (III) *vs.* T2N1 (IIB)	14.9 m (95%CI: 13.1–16.7 m) *vs.* 15.1 m (95%CI: 14.3–15.9 m)	0.481
–	T1N2 (III) *vs.* T3N0 (IIA)	14.9 m (95%CI: 13.1–16.7 m) *vs.* 15.5 m (95%CI: 14.6–16.4 m)	0.210
–	T2N1 (IIB) *vs.* T3N0 (IIA)	15.1 m (95%CI: 14.3–15.9 m) *vs.* 15.5 m (95%CI: 14.6–16.4 m)	0.445
