# Supplementary material for: Validation of the eighth edition of the AJCC staging system for patients with pancreatic adenocarcinoma initially receiving chemoradiotherapy and proposal of modifications
Source: Cancer Biol Med. 2020 May 15;17(2):492–500. doi: 10.20892/j.issn.2095-3941.2019.0101 (PMC7309473; doi:10.20892/j.issn.2095-3941.2019.0101)
Supplement: Supplementary file 1 [file cbm-17-492-s001.pdf]

Supplementary materials

Table S1 Cross tabulation of the seventh and eighth editions of the staging system

Seventh edition	Eighth edition, *n* (%)					
	IA	IB	IIA	IIB	III	Total
IA	10 (1.5)	0	0	0	0	10 (1.5)
IB	0	79 (11.6)	18 (2.6)	0	0	97 (14.2)
IIA	0	22 (3.2)	119 (17.4)	0	0	141 (20.6)
IIB	0	0	0	154 (22.5)	112 (16.4)	266 (38.9)
III	0	0	0	0	169 (24.7)	169 (24.7)
Total	10 (1.5)	101 (14.8)	137 (20.1)	154 (22.5)	281 (41.1)	683 (100.0)

Table S2 Baseline characteristics of patients with tumor invasions of the portal vein with or without tumor thrombi, and T4 tumors, according to the eighth edition

Characteristics	Patients with tumor invasions of the portal vein with or without tumor thrombi, *n* (%)	Patients with T4 tumors, *n* (%)	*P*
Number of patients	92	169	
Age, years, median (range)	68 (38–87)	66 (39–86)	0.102
Gender			
Male	55 (59.8%)	104 (61.5%)	0.781
Female	37 (40.2%)	65 (38.5%)	
Tumor diameter (cm), median (range)	4.1 (2–8.8)	3.9 (2–8.6)	0.451
N stage			
N0	44 (47.8%)	75 (44.4%)	0.354
N1	40 (43.5%)	69 (40.8%)	
N2	8 (8.7%)	25 (14.8%)	
Prescription dose (Gy), median (range)	37 (30–44)/5-8f	36 (30–46.8)/5-8f	0.664
BED10 (Gy), median (range)	61.92 (48–82.72)/5-8f	61.92 (48–85.5)/5-8f	0.385

Table S3 Patient characteristics of the cohort from the SEER database

Characteristics	*n* (%)
No. of patients	176
Age, years	
< 65	85 (48.3)
≥ 65	91 (51.7)
Gender	
Male	94 (53.4)
Female	82 (46.6)
TNM stage, 7th edition	
IA	4 (2.3)
IB	10 (5.7)
IIA	49 (27.8)
IIB	35 (19.9)
III	78 (44.3)
TNM stage, 8th edition	
IA	10 (5.7)
IB	53 (30.1)
IIA	14 (7.9)
IIB	20 (11.4)
III	79 (44.9)

Table S4 Survival outcomes of the cohort from the SEER database, according to the eighth edition and modified staging system

		Eighth edition	*P*	Modified staging system	*P*
		OS (95%CI)		OS (95%CI)	
Cancer stage	IA	21.0 m (95%CI: 6.5–35.5 m)	< 0.001	21.0 m (95%CI: 6.5–35.5 m)	< 0.001
	IB	17.0 m (95%CI: 15.5–18.5 m)		17.0 m (95%CI: 15.0–19.0 m)	
	IIA	12.0 m (95%CI: 0–24.4 m)		15.0 m (95%CI: 11–19.0 m)	
	IIB	13.0 m (95%CI: 10.4–15.5 m)		10.0 m (95%CI: 8.2–11.8 m)	
	III	8.0 m (95%CI: 6.2–9.8 m)		IIIA: 9.0 m (95%CI: 5.3–12.7 m)	
	–	–		IIIB: 8.0 m (95%CI: 3.9–12.1 m)	

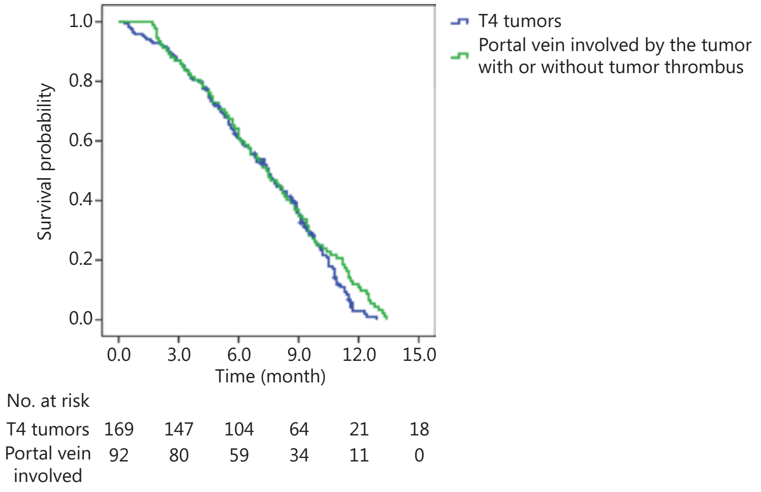

Figure S1 OS of patients with PV ± PVTT and patients with T4 tumors.

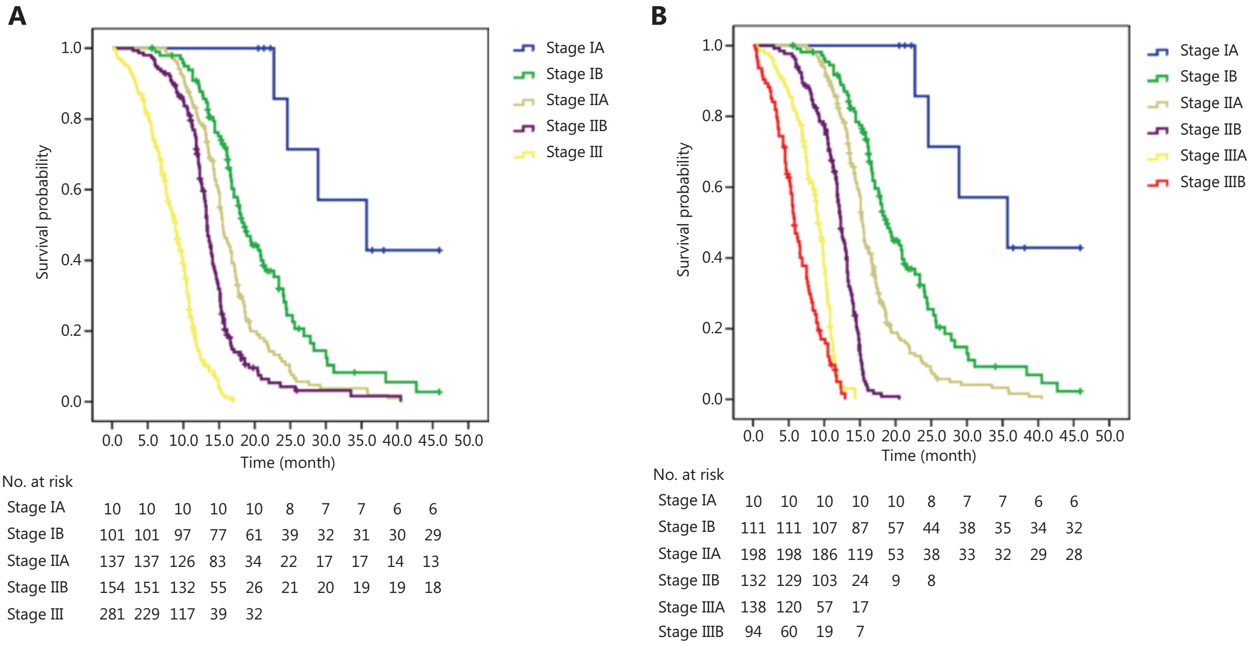

Figure S2 (A) Patient OS according to the eighth edition and (B) the modified staging system.

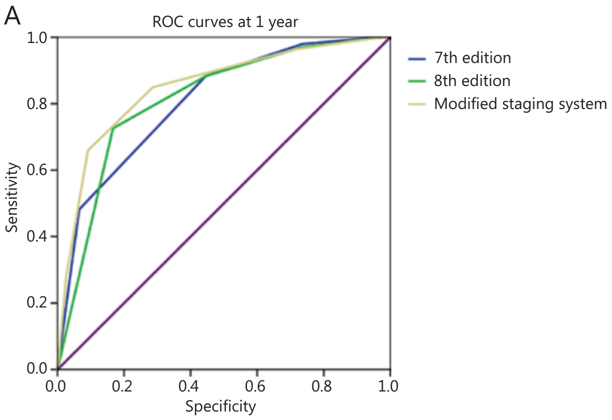

Figure S3 (A) ROC curves according to the seventh and eighth editions, and the modified staging system at 1 year after treatment and (B) 2 years after treatment.
